# Discrimination of a single-item scale to measure intention to have a COVID-19 vaccine

**DOI:** 10.1371/journal.pone.0322503

**Published:** 2025-05-05

**Authors:** Julius Sim, Louise E. Smith, Richard Amlôt, G. James Rubin, Nick Sevdalis, Susan M. Sherman

**Affiliations:** 1 School of Medicine, Keele University, Keele, United Kingdom; 2 Behavioural Science and Insights Unit, United Kingdom Health Security Agency, London, United Kingdom; 3 NIHR Health Protection Research Unit in Emergency Preparedness and Response, King’s College London, London, United Kingdom; 4 Institute of Psychiatry, Psychology and Neuroscience, King’s College London, London, United Kingdom; 5 Centre for Behavioural and Implementation Science Interventions, Yong Loo Lin School of Medicine, National University of Singapore, Singapore, Singapore; 6 School of Psychology, University of Sheffield, Sheffield, United Kingdom; Sapienza University of Rome, ITALY

## Abstract

**Aim:**

When developing public health measures in a pandemic, it is important to examine attitudes and beliefs relating to vaccination uptake. We report the discrimination of a single-item vaccination intention scale and derive cutpoints in terms of sensitivity (true positives) and specificity (true negatives) in relation to subsequent vaccination status.

**Subject and Methods:**

In a sample of UK adults (*n*=1119) recruited through an online survey platform, vaccination intention was measured on a 0–10 numerical rating scale (0=very unlikely, 10=very likely) at the beginning of the UK COVID-19 vaccination rollout (January 2021), and self-reported vaccination status was gathered after vaccination had been offered to all adults (October 2021). Discrimination of the scale was measured by the area under the receiver operating characteristic (ROC) curve.

**Results:**

The responders reporting being vaccinated or unvaccinated were 1034 (92.4%) and 85 (7.6%), respectively. The area under the ROC curve was.956 (95% CI.943,.967), indicating a high degree of discrimination. The combined value of sensitivity and specificity was greatest at a cutpoint of 8 on the scale (sensitivity =.821, specificity =.988). If, however, the individual values of sensitivity and specificity are required to be simultaneously optimized, this occurs at point 6 (sensitivity =.886, specificity =.871).

**Conclusion:**

We recommend a 0–10 intention scale as a validated, practical measure of vaccination intention in public health practice, with a cutpoint of 8 on the scale as optimal, unless sensitivity and specificity are to be simultaneously optimized, when 6 is the optimal cutpoint.

## Introduction

Effective public health measures are crucial in countering the impact of a pandemic such as COVID-19. It is important, therefore, to understand attitudes and beliefs in the general public towards outbreaks of infectious diseases, and intentions regarding the uptake of specific preventive initiatives in particular, as the basis for an appropriate and effective public health strategy [1]. A key element in such a strategy in relation to COVID-19 has been vaccination [2,3]. A number of recent studies have focused on sociodemographic, clinical, behavioural and attitudinal factors associated with willingness to be vaccinated against COVID-19 and have included a measure of vaccination intention [4–8]. These studies attempt to identify psychological factors that could be modified and socio-demographic variables that could be targeted to improve vaccination uptake. However, intention does not always lead to behaviour [9].

Multi-item measures of vaccination intention potentially have good predictive properties by virtue of their coverage of multiple predictors, and the use of multiple items increases the reliability (internal consistency) of a scale [10]. There is, however, an abundance of multi-item scales investigating vaccine- or theory-specific determinants of vaccination through self-report. The sheer volume and variability of those scales means that a single-item measure might be helpful not only in offering an efficient (if imperfect) measure, but also in allowing some comparability if used alongside and/or correlated with multi-item scales in subsequent research. Additionally, single-item measures may be more practical in routine clinical or public health practice, where the time and resources required to collect and analyse data from a wide range of variables may be lacking. Similarly, single-item measures may be more suitable for inclusion in population surveys that seek to cover a broad range of attitudes, beliefs or reported behaviours, where parsimony may be needed in terms of the items included in the survey instrument and the amount of data generated [11]. Single-item measures may also be more intuitive to respondents, thereby increasing their face validity; this, and the speed with which a single item can be completed, may minimize missing data and improve the response rate [12]. Furthermore, whilst the determinants of a person’s intention to be vaccinate are likely to be multiple, requiring separate indicators of different constructs, the strength of the resulting intention is unidimensional and thus in principle suited to measurement by a single item.

Attitudes and related psychological variables are typically measured on some type of numerical rating scale, so that their intensity can be quantified. If, however, a rating scale is used to indicate the intended performance or non-performance of a future action, an appropriate cutpoint has to be identified on the scale in order to derive this binary classification. Such a cutpoint should demonstrate good discrimination (classificatory accuracy). Using data from a broader study, we report on the discrimination of a simple, pragmatic single-item rating scale used to measure intention to have a COVID-19 vaccination in a UK sample of adults and derive an optimal cutpoint on the scale.

The aim of this study was to test the discrimination of a single-item vaccination intention scale, and to derive cutpoints, in terms of sensitivity and specificity, that could act as practical indicators of future vaccination status.

### Statistical considerations

In clinical epidemiology, important statistical features of a test intending to produce a diagnostic, prognostic or other binary classification are sensitivity and specificity. These statistics indicate the relationship between a positive or negative outcome on the test and the true positive or negative status of those tested. Sensitivity indicates the proportion of those who test positive that are truly positive and thereby indicates the probability of a positive test given that disease is present, while specificity indicates the proportion of those who test negative that are truly negative and thereby indicates the probability of a negative test given that disease is absent [13]. They are estimated as [14]:

$\text{Sensitivity =}\frac{\text{true negatives}}{\text{true negatives + false positives}}$

$\text{Specificity =}\frac{\text{true negatives}}{\text{true negatives + false positives}}$

There is a trade-off between sensitivity and specificity – improving the sensitivity of a test will reduce its specificity, and vice versa. Sensitivity and specificity are not influenced by the prevalence of the outcome of interest.

It is also possible to calculate positive and negative predictive value. Whereas sensitivity is the probability of a positive test when disease is present, positive predictive value is the probability of disease when a test is positive. Correspondingly, whereas specificity is the probability of a negative test when disease is absent, negative predictive value is the probability of the absence of disease when a test is negative. Unlike sensitivity and specificity, positive and negative predictive value are influenced by the prevalence of the disease [15].

Translating this into the current case, being classified as positive or negative equates to being deemed likely or unlikely to be vaccinated, respectively, and being a true positive or a true negative equates to subsequently being vaccinated or unvaccinated, respectively (Table 1). The sensitivity and specificity of a specific cutpoint on an attitude scale used to predict vaccination status can be calculated accordingly.

**Table 1:** Relationship between predictive classification from the intention scale and subsequent vaccination status.

		Subsequent vaccination status	
		Vaccinated	Unvaccinated
**Classification derived from scale**	Likely	True positive	False positive
	Unlikely	False negative	True negative

## Materials and methods

### Sample

Data were obtained from two waves of the COVID-19 Vaccine Acceptability Study (CoVAccS), which investigated attitudes and beliefs relating to COVID-19 and COVID-19 vaccination in the UK; measurement of vaccination intention was part of this study. The survey recruited a representative sample of respondents through an online survey research platform (Qualtrics); detailed methods are reported elsewhere [16,17]. One wave of data collection was completed during 13–15 January 2021, at the very beginning of the UK COVID-19 vaccination rollout (T1), with a further longitudinal follow-up wave completed during 4–15 October 2021, after the vaccination had been offered to all adults (T2). The T1 questionnaire included a question on vaccination intention: ‘Now that a coronavirus vaccination is available, how likely is it that you will have one?’ Participants were asked to respond on an eleven-point numerical rating scale, anchored 0 = ‘extremely unlikely’ and 10 = ‘extremely likely’ (S1 Fig). A continuous, rather than a categorical, intention scale had been used in the main study in order to facilitate the analyses used in a linear regression model.

Of the 1148 respondents to both waves of the survey, five had missing data and those who had already received one or more doses of the vaccine at T1 (*n*=24) were excluded from the analysis. This left 1119 participants (97.5%) who had completed the intention scale at T1 and subsequently reported their vaccination status at T2. The mean (standard deviation) age of participants at T1 was 48.2 (15.1) years. There were 594 (53.1%) female and 522 (46.6%) male respondents; 3 (0.3%) other gender identities were reported.

### Statistics

The discrimination of the intention scale and an appropriate predictive cutpoint within the scale were identified through nonparametric receiver operating characteristic (ROC) analysis. This produces a ROC curve that plots sensitivity (true positive rate) on the vertical axis against 1 – specificity (false positive rate) on the horizontal axis. A curve that assumes a straight line on an upward diagonal from the bottom left-hand to the top right-hand corner on this plot represents a process of classification that is no better than chance, or guessing. Curves that depart from the diagonal and approach the upper left-hand corner represent progressively greater discrimination. Movement of the curve upwards on the vertical axis denotes increasing sensitivity and movement to the left on the scale denotes increasing specificity, i.e., lower values of (1 – specificity). The area under the ROC curve (AUC) was estimated and an exact binomial 95% confidence interval (CI) calculated. The AUC quantifies the classificatory accuracy of the scale, taken across the full range of cutpoints, from a minimum of 0 to a maximum of 1 [13]. The points on the scale can be identified at different places on the ROC curve and their respective discrimination can thereby be determined.

A cutpoint was defined as the point on the scale *at or above* which an individual would be predicted to be vaccinated. We examined two methods that have been proposed to identify an optimal cutpoint. The first of these, the Youden index [18], represents the point on the scale at which sensitivity and specificity are maximized, without according any differential weight to either sensitivity or specificity – it seeks the overall maximum of the two. This index is calculated for a specific cutpoint (*c*) as:

$J_{c}=\text{sensitivit}{\text{y}}_{c}+\left(\text{specificit}{\text{y}}_{c}-1\right)$

For example, a cutpoint with a sensitivity.610 of and a specificity of.720 would yield a Youden index of.610 + (.720–1) =.330. The point on the scale with the largest Youden index is the optimal cutpoint on this criterion.

The second method is the index of union [19], which differs from the Youden index in that it seeks to maximize sensitivity and specificity at the same time; it seeks the point on the scale at which both of these are simultaneously as high as possible. This statistic takes into account the AUC from the ROC curve and is calculated for a specific cutpoint (*c*) as:

$IU_{c}=\left(\left|\text{sensitivit}{\text{y}}_{c}-\text{AUC}\right|+\left|\text{specificit}{\text{y}}_{c}-\text{AUC}\right|\right)$

For example, if the AUC were.789, a cutpoint with a sensitivity.610 of and a specificity of.720 would yield an index of union of (│610 –.789│ + │.720 –.789│) =.248. The point on the scale with the smallest index of union index is the optimal cutpoint on this criterion.

Finally, we calculated positive likelihood ratios (LR +) and negative likelihood ratios (LR –) for points on the scale [20]:

LR+ = sensitivity /(1−specificity)

The positive likelihood ratio is the probability of a positive classification among vaccinated individuals divided by the probability of a positive classification among unvaccinated individuals. A ratio greater than 1 indicates how much more likely a vaccinated individual is to be classified as positive than an unvaccinated individual; higher values are better. The negative likelihood ratio is the probability of a negative classification for a vaccinated individual divided by the probability of a negative classification for an unvaccinated individual. A ratio less than 1 indicates how much less likely a vaccinated individual is to be classified as negative than an unvaccinated individual; lower values are therefore better. Analyses were conducted in Stata 17.

### Ethics

Ethical approval for this study was granted by Keele University Research Ethics Committee (reference: PS-200129). Written **c**onsent was obtained from all participants in the study.

## Results

**Fig 1:**
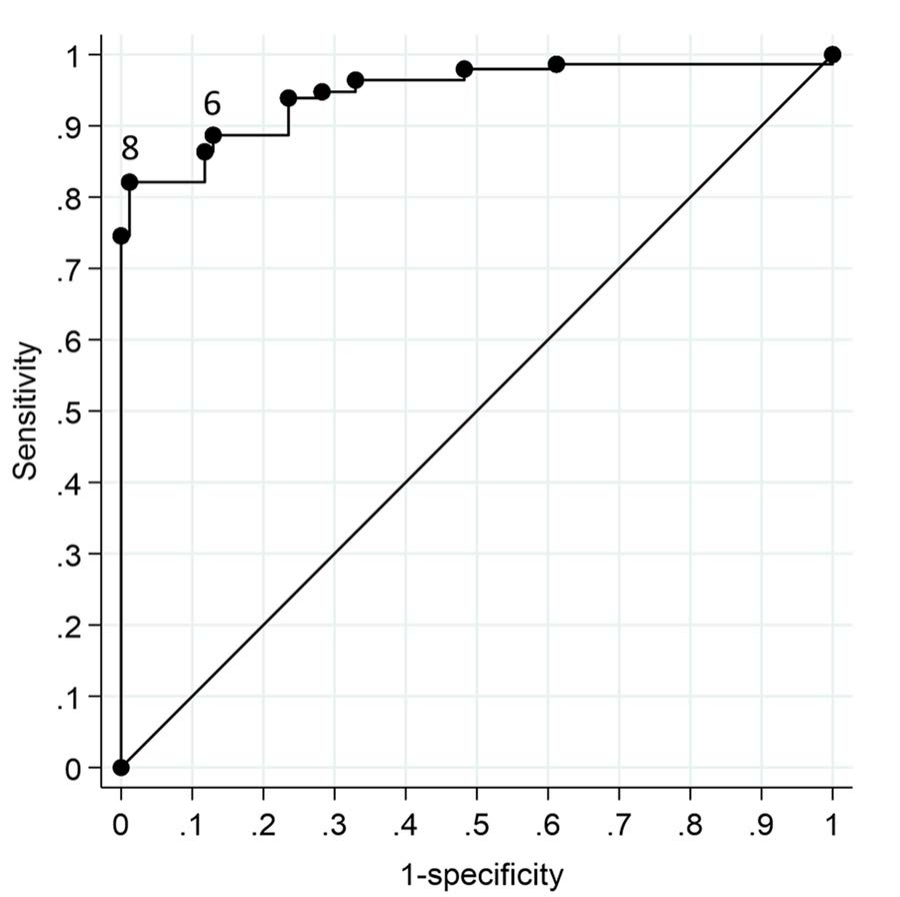
ROC curve for the intention scale, showing sensitivity (true positive rate) and 1–specificity (false positive rate). Points 6 and 8 on the scale are indicated.

Table 2 indicates, for each point on the intention scale, the number of participants who reported being either vaccinated or unvaccinated. Of the 1034 (92.4%) participants who reported being vaccinated, 25 reported having received one dose and 1009 reported having received two doses. The vaccine received was reported as follows: Astra-Zeneca, 597 (57.7%); Pfizer-BioNtech, 395 (38.2%); Moderna, 36 (3.5%); Janssen (Johnson & Johnson), 1 (0.1%); another vaccine or don’t know, 5 (0.5%). Only 85 (7.6%) out of 1119 respondents remained unvaccinated; none of these had scored either 9 or 10 on the intention scale. Fig 1 shows the ROC curve. The AUC was.956 (95% CI .943,.967; *n*=1119), indicating a high degree of discrimination.

**Fig 2:**
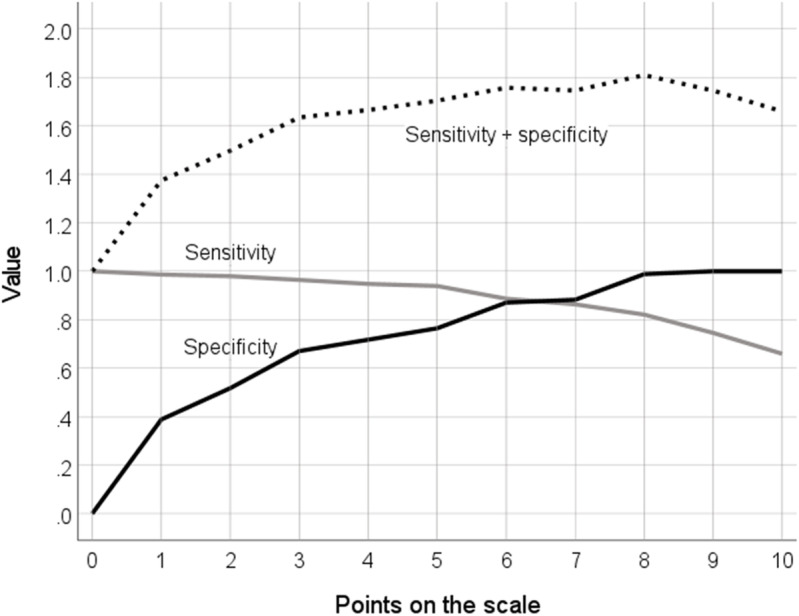
Values of sensitivity, specificity, and sensitivity-plus-specificity for points on the intention scale.

Table 3 shows the sensitivity and specificity of each point on the scale, as well as the likelihood ratios, the Youden index and the index of union. Values of sensitivity, specificity, and sensitivity-plus-specificity are also displayed in Fig 2. The maximum combined sensitivity and specificity was for point 8 on the scale, as it here that the Youden index was highest (.809). Here, sensitivity is .821 (95% CI .797, .843) and specificity is .988 (95% CI .936, .998). The positive likelihood ratio for this point on the scale is 69.792, indicating that a person ultimately vaccinated is nearly 70 times more likely to be classified as positive than a person ultimately unvaccinated, at this cutpoint. The negative likelihood ratio of 0.181 for point 8 indicates that a person ultimately vaccinated is only 18% as likely to be classed as negative than a person ultimately unvaccinated. At point 8, positive predictive value is .999 (95% CI .993, 1.000) and negative predictive value is .312 (95% CI .260, .370).The lowest index of union (.154) is for scale point 6, indicating that sensitivity and specificity are simultaneously at their maximum at this point; sensitivity is .886 (95% CI .865, .904) and specificity is .871 (95% CI .783, .926). Here, positive predictive value is .988 (95% CI .979, .993), and negative predictive value is .387 (95% CI .321, .458).

**Table 3:** Statistics relating to each point on the intention scale. The optimal (i.e., largest) Youden index and the optimal (i.e., smallest) index of union are in bold.

			Likelihood ratios		Indices		Predictive value	
Scale point	Sensitivity	Specificity	Positive	Negative	Youden index	Index of union	Positive	Negative
0	1.00	0.000	1.000	–				
1	.987	.388	1.613	0.035	.375	.598	.952	.702
2	.980	.518	2.031	0.039	.498	.462	.961	.677
3	.964	.671	2.927	0.053	.635	.293	.973	.606
4	.948	.718	3.357	0.073	.666	.246	.976	.530
5	.939	.765	3.991	0.080	.704	.208	.980	.508
6	.887	.871	6.853	0.130	.758	**.154**	.988	.387
7	.864	.882	7.341	0.155	.746	.166	.989	.347
8	.821	.988	69.792	0.181	**.809**	.167	.999	.312
9	.746	1.000	–	0.254	.746	.254	1.000	.244
10	.660	1.000	–	0.340	.660	.340	1.000	.195

**Table 2:** Number (row percentage) of participants ultimately vaccinated or unvaccinated, for each point on the intention scale.

Point on scale	Vaccination status		
	Unvaccinated	Vaccinated	Total
0	33 (70.2)	14 (29.8)	47
1	11 (61.1)	7 (38.9)	18
2	13 (44.8)	16 (55.2)	29
3	4 (19.0)	17 (81.0)	21
4	4 (30.8)	9 (69.2)	13
5	9 (14.3)	54 (85.7)	63
6	1 (4.0)	24 (96.0)	25
7	9 (17.0)	44 (83.0)	53
8	1 (1.3)	78 (98.7)	79
9	0 (0.0)	89 (100.0)	89
10	0 (0.0)	682 (100.0)	682
Total	85	1034	1119

One might wish to use the intention scale to predict unvaccinated, rather than vaccinated, status, defined as a scale point *at or below* which an individual is predicted to remain unvaccinated. The overall discrimination of the scale and the optimal cutpoints remain unchanged, but values of sensitivity and specificity are transposed. These are shown in in Table 4.

**Table 4:** Statistics relating to each point on the intention scale where ‘unvaccinated’ is the classification of interest.

Scale point	Sensitivity	Specificity
0	.388	1.000
1	.518	.987
2	.671	.980
3	.718	.964
4	.765	.948
5	.871	.939
6	.882	.887
7	.988	.864
8	1.000	.821
9	1.000	.746
10	1.000	.660

## Discussion

Our analysis suggests that an optimal cutpoint on this intention scale in relation to a judgment of subsequent vaccination is 8, in that the combination of sensitivity and specificity is greater here than at any other point on the scale. This is the *a priori* cutpoint that had been used in previous analysis of data from the CoVAccS study [16,17,21] and our analysis here validates this choice. If, however, it is desired to maximize both sensitivity and specificity simultaneously, the optimal cutpoint would be 6, as both sensitivity and specificity are at their maximum at this point on the scale.

The Youden index and the index union serve to maximize sensitivity and specificity, in different ways. However, there may be reasons to opt for cutpoints that prioritize one of the other of these statistics. Accordingly, one can choose other cutpoints on the scale with a view to achieving a high level of either sensitivity or specificity, based upon the relative costs of misclassification (i.e., false positives or false negatives). For example, it might be thought most important to avoid a high false negative rate, which would mean that the future vaccination rate would be underestimated and efforts to promote vaccination might therefore be disproportionate to their benefit. In such a scenario, the emphasis would be placed on sensitivity and a cutpoint lower than 8 might be favoured (Table 3). However, this requires caution in view of the inverse relationship of sensitivity and specificity; privileging sensitivity in this way will be at the cost of specificity. Conversely, the concern might be about a high false positive rate, whereby the future vaccination rate would be overestimated and insufficient effort might be put into a vaccination campaign as a result. Here, the emphasis would switch to specificity and a cutpoint of 8, at which specificity is virtually at its maximum, would remain a reasonable choice, though at the cost of some degree of sensitivity.

There are differing opinions on what constitutes a poor, moderate or good value of the AUC, but according to de Hond et al [22], most commentators would classify the value of.956 that we obtained under the labels ‘very good’, ‘high’ or ‘excellent’. Our estimate compares favourably with the findings of a study examining vaccination willingness, as predicted by a measure of fear of COVID-19; this yielded AUC values of.57 and.69, at two different timepoints [23]. Other studies of prediction tools related to COVID-19 have yielded AUC values of.79 [24],.88 [25] and.92 [26], with which our estimate again compares favourably.

A strength of our analysis is that participants’ reporting of vaccination status is highly unlikely to have been influenced by recall of their prior vaccination intention as this occurred more than eight months previously. The sample size was large and therefore provided a precise estimate of the AUC, as indicated by the width of the associated confidence interval. However, the small number of unvaccinated participants means that our estimates of specificity in Table 3 are less precise than those of sensitivity (and, correspondingly, our estimates of sensitivity in Table 4 are less precise than those of specificity). It should be noted that our analysis is based on reported vaccination status rather than actual vaccination status, on which we had no information. It is unlikely that participants wrongly recalled their vaccination status [27–30], but they might have reported it incorrectly through the influence of social desirability or cognitive dissonance. As the study was completed anonymously, the influence of social desirability should be lessened. It is also possible that some respondents confused vaccination for COVID-19 with vaccination for influenza, though as we asked respondents separately about influenza vaccination this may be unlikely. The use of projective techniques [31] or other indirect measures of intention may offset a social desirability bias, but may be less intuitive than a question directly focused on vaccination intention.

Positive and negative predictive value depend upon prevalence, and in a population with a lower rate of vaccination, positive predictive value would be expected to be lower, and negative predictive value to be higher, than reported here [15]. The estimates of these statistics that we have reported should not therefore be generalized to populations in which the rate of vaccination differs. In contrast, sensitivity and specificity do not depend upon prevalence, so our findings may apply to other countries with different rates of vaccine uptake. However, further testing and validation would be required to determine whether they are transferable to other populations, or to other types of vaccination than COVID-19. Differences in the perceived risk of a different type of infection and of the effectiveness of the vaccine available, together with public health messaging and the availability of relevant healthcare interventions, might affect the measurement of vaccination intention. Finally, our findings relate to vaccination status nine months after vaccination intention was measured, as dictated by the broader study in which this analysis is based; those who were unvaccinated at this time may well have subsequently received a vaccine.

## Conclusion

A single-item measure of vaccination intention is quick to administer and does not require complex or lengthy analysis in order to predict subsequent (non-)uptake of vaccination. In view of the good discrimination of the 0–10 vaccination intention scale (with anchors of ‘extremely unlikely’ and ‘extremely likely’) that we have evaluated, its use represents an effective yet pragmatic option within routine public health practice to identify individuals who will subsequently have themselves vaccinated. Unless a differential weight is to be placed on either sensitivity or specificity, we recommend a cutpoint of 8 when seeking to identify those who will subsequently receive vaccination.

## Supporting information

S1 Fig Single-item vaccine intention scale. (PDF)
