# Supplementary material for: Discrimination of a single-item scale to measure intention to have a COVID-19 vaccine
Source: PLoS One. 2025 May 5;20(5):e0322503. doi: 10.1371/journal.pone.0322503 (PMC12052102; doi:10.1371/journal.pone.0322503)
Supplement: S1 Fig — (PDF) [file pone.0322503.s001.pdf]

The single-item vaccination intention scale:

When a coronavirus vaccination becomes available to you, how likely is it that you will have one? Please select a number between 0 and 10, where **0 means ‘extremely unlikely’ and 10 means ‘extremely likely’**:

extremely unlikely										extremely likely
0	1	2	3	4	5	6	7	8	9	10
○	○	○	○	○	○	○	○	○	○	○
